# Concomitant decrease of E- and A-FABP expression predicts worse survival in urothelial bladder cancer patients

**DOI:** 10.1038/s41598-024-65972-8

**Published:** 2024-07-04

**Authors:** Inès Saizonou, Isabelle Lascombe, Franck Monnien, Isabelle Bedgedjian, François Kleinclauss, Marie-Paule Algros, Sylvie Fauconnet

**Affiliations:** 1 CHU Besançon, Service Anatomie et Cytologie Pathologiques, 25000 Besançon, France; 2 Université Franche-Comté, SINERGIES – LabEx LipSTIC ANR-11-LABX-0021, 25030 Besançon, France; 3 CHU Besançon, Service Urologie, Andrologie et Transplantation Rénale, 25000 Besançon, France; 4 CHU Besançon, Centre Investigation Clinique, Inserm CIC 1431, 25000 Besançon, France

**Keywords:** Tumour biomarkers, Urological cancer

## Abstract

Non-muscle invasive bladder cancers (NMIBC) pTa-pT1 are depicted by a high risk of recurrence and/or progression with an unpredictable clinical evolution. Our aim was to identify, from the original resection specimen, tumors that will progress to better manage patients. We previously showed that A-FABP (Adipocyte- Fatty Acid Binding Protein) loss predicted NMIBC progression. Here we determined by immunohistochemistry the prognostic value of E-FABP (Epidermal-Fatty Acid Binding Protein) expression in 210 tumors (80 pTa, 75 pT1, 55 pT2-T4). Thus, E-FABP low expression was correlated with a high grade/stage, the presence of metastatic lymph nodes, and visceral metastases (*p* < 0.001). Unlike A-FABP in NMIBC, E-FABP low expression was not associated with RFS or PFS in Kaplan–Meier analysis. But patients of the overall cohort with a high E-FABP expression had a longer mOS (53.8 months vs. 29.3 months, *p* = 0.029). The immunohistochemical analysis on the same NMIBC tissue sections revealed that when A-FABP is absent, a high E-FABP expression is detected. E-FABP could compensate A-FABP loss. Interestingly, patients, whose original tumor presents both low E-FABP and negative A-FABP, had the worse survival, those maintaining the expression of both markers had better survival. To conclude, the combined evaluation of A- and E-FABP expression allowed to stratify patients with urothelial carcinoma for optimizing treatment and follow-up.

## Introduction

Bladder cancer comprises 3% of all diagnosed cancers in the world, with about 550,000 new cases in 2018 and 200,000 leading cause of death^1^. Over 90% of bladder cancers originate from the urothelium^2^. At the time of diagnosis, 70–80% of patients present a non-muscle invasive bladder cancer (NMIBC) and 20–30% present a muscle invasive bladder cancer (MIBC) or a metastatic disease. NMIBC comprise carcinoma in situ (C*is*), papillary tumors confined to the mucosa (pTa) or invading the lamina propria (pT1) but not the muscularis propria^3^. Their management includes transurethral resection (TUR) with or without endovesical chemotherapy. However, despite TUR and intravesical treatments, 50–80% of pTa present a high risk of recurrence and a low risk of progression to a muscle-invasive disease (5%). In contrast, pT1 tumors are very heterogeneous and characterized by a high risk of progression. Thus, 30% pT1 with high grade/G3 or associated C*is* will progress to MIBC or metastatic disease generally with an unfavorable prognosis (5-year survival < 50%)^4–7^. For MIBC, the radical cystectomy is the standard treatment^8,9^. In this context, the identification of pT1 tumors that will progress is necessary for therapeutic decision-making. Clinical staging and histopathological parameters are not sufficient to characterize the tumor behavior. Thus, an accurate prediction of progression, defined as a disease recurrence with an increase in grade, stage or metastatic status from the original diagnosis, is critically important for the management of NMIBC and is crucial to determine appropriate therapy and follow-up. That is why, the research of biomarkers to identify patients at high risk of recurrence and progression is required.

In a previous study, we demonstrated that loss of A-FABP (Adipocyte-Fatty Acid Binding Protein/FABP4/aP2) expression was correlated with high tumor stage and high histological grade in UC More precisely, the weak expression of A-FABP in resected primary NMIBC group was a higher risk factor of progression associated with poorer prognosis^10^. A-FABP is a member of the FABP (fatty acid binding protein) family. FABPs are small proteins (14–15-kDa) known as intracellular lipid chaperons that regulate fatty acid (FAs) uptake, transport and trafficking. They can reversibly bind saturated and unsaturated long-chain FAs as well as eicosanoids. Therefore, FABPs may be involved in the metabolic regulation of FAs during the process of carcinogenesis given that FAs serve oncogenic roles in the majority of cancer types. In fact, abnormal expression and function of FABPs could be involved in tumorigenesis^11^. Among members of the FABP family, E-FABP (Epidermal-FABP/FABP5/keratinocyte-FABP) has been reported to be upregulated in some cancers (prostate, breast) and to participate in cancer progression^11^. In contrast, a study showed that less-differentiated bladder tumors were characterized by a decreased expression of E-FABP protein^12^. Interestingly, it has been reported that in adipose tissue of A-FABP^-/-^ mice, the amount of E-FABP was increased^13^ compared with control mice. It is noted that A-FABP and E-FABP have 52% amino acid similarity and bind several FAs with similar affinity and selectivity^14^. These results suggested a same function of both proteins and thus a compensatory role of E-FABP to maintain a normal physiology in A-FABP-deficient mice.

After A-FABP our interest focused on E-FABP also because they are the only members of the FABP family expressed in bladder cancers. The aim of the present study was to investigate by immunohistochemistry the expression of E-FABP in a cohort of 210 patients with urothelial carcinoma (UC) of different stages (pTa, pT1, pT2-T4) and grades (PUNLMP, low grade, high grade) to evaluate whether it could be associated with clinicopathological parameters (grade, stage, …), and it could help predict disease recurrence and or/progression in pTa and pT1 patient groups after TUR.

To our knowledge this is the only immunohistochemical study of E-FABP expression in urothelial carcinomas. Furthermore, since loss of A-FABP is an indicator of tumor progression in NMIBC, we analyzed in the same cohort of patients the status of E-FABP and A-FABP and we interested in E-FABP level when A-FABP expression was decreased. Specifically, we asked whether a high E-FABP level could be associated with the loss of A-FABP and whether the simultaneous study of the two biomarkers is more informative than the analysis of a single marker. E- and A-FABP expression level was also analyzed on overall tumors to make OS curves.

## Materials and methods

### Patient cohort and tumor material

In this retrospective study, all eligible patients were diagnosed for bladder UC in the Pathology Department of Jean Minjoz University Hospital (Besançon, France) from January 2000 through January 2016. The follow-up period for all patients was defined as the interval between the date of operation and the date of April 2020. We included 210 patients with an initial diagnosis of bladder cancer who underwent operations (TUR, radical cystectomy). We excluded cases of kidney, prostate or ureter cancers associated with bladder cancer and patients without clinical information or usable samples.

All tumors were from initial presentation of bladder UC and none of the patients, from whom the samples were retrieved, had received chemotherapy or radiotherapy prior to surgery. Histology, grade and stage were determined by pathologic examination of the transurethral resection or cystectomy and were confirmed by blinded re-review of the original cystoscopic biopsy slides. Tumors were staged according to the 2017 Tumor-Node-Metastasis (TNM) staging system guidelines and graded according to the 2004 WHO/ISUP (World Health Organization/International Society of Urological Pathology) classification. Patient characteristics, pathological type of all specimens (80 pTa, 75 pT1 and 55 ≥ pT2-4) and clinicopathologic parameters are described in Table 1. For pTa and pT1 tumor group analysis, tumor recurrence was defined, after transurethral resection, as the reappearance of the tumor in the bladder at a lower or equivalent pathological stage or grade and tumor progression was characterized by a recurrence of the bladder tumor with an increase in grade or stage from the original tumor. The pT2-T4 tumors were only used to determine the differential expression level of A- and E-FABP in the different stages. They were not used for the follow-up of the patients except to make OS curves.

**Table 1:** Clinicopathological data according to E- and A-FABP expression.

Parameters	N (%)	Low E-FABP (≤ 30%), n (%)	High E-FABP (> 30%), n (%)	*p* value	Negative A-FABP (≤ 10%) n (%)	Positive A-FABP (> 10%) n (%)	*p* value
Population	210	47 (22.38)	163 (77.62)		128 (60.95)	82 (39.05)	
Sex							
Male	171 (81.43)	39 (22.80)	132 (77.20)	0.834	106 (61.98)	65 (38.01)	0.64
Female	39 (18.57)	8 (20.51)	31 (79.48)		22 (56.41)	17 (43.59)	
Age							
Mean (sd)	70.7 (12.9)	72.2 (13.80)	70.5 (12.70)	0.435	71.3 (12.10)	70.3 (14.20)	0.46
Histology							
Papillary	191 (91.38)	40 (20.94)	151 (79.05)	0.134	113 (59.16)	78 (40.83)	0.08
Variants*	18 (8.62)	7 (38.88)	11 (61.11)		15 (83.33)	3 (16.66)	
pT							
pTa	80 (38.10)	5 (6.25)	75 (93.75)	< 0.001	34 (42.50)	46 (57.50)	< 0.001
pT1	75 (35.71)	17 (22.67)	58 (77.33)		43 (57.33)	32 (42.66)	
pT2-T4**	55 (26.19)	25 (45.45)	30 (54.54)		51 (92.72)	4 (7.27)	
pN							
0	183 (87.14)	33 (18.03)	150 (82.00)	< 0.001	104 (56.83)	79 (43.17)	< 0.01
1–2	27 (12.86)	14 (51.85)	13 (48.15)		24 (88.9)	3 (11.11)	
pM							
0	193 (91.90)	37 (19.17)	156 (80.83)	0.001	113 (58.55)	80 (41.45)	0.03
1	17 (8.10)	10 (58.82)	7 (41.17)		15 (88.23)	2 (11.76)	
Grade (WHO/ISUP 2004)							
PUNLMP	20 (9.52)	1 (5.00)	19 (95.00)	< 0.001	5 (25.00)	15 (75.00)	< 0.001
LG-UC	60 (28.57)	4 (6.67)	56 (93.33)		31 (51.66)	29 (48.33)	
HG-UC	130 (61.91)	42 (32.30)	88 (67.70)		92 (70.77)	38 (29.23)	
C*is*							
Yes	10 (4.78)	4 (40.00)	6 (60.00)	0.237	6 (60.00)	4 (40.00)	1
No	199 (95.22)	43 (21.60)	156 (78.40)		122 (61.30)	77 (38.70)	

*The histological variants include micropapillary, nested, reversed and sarcomatoid variants, urothelial carcinoma with squamous, glandular or neuroendocrine differentiation. **Due to some reduced numbers, the pT3 and pT4 stages were grouped together with the pT2 stage for the Khi-2 test. *PUNLMP*, papillary urothelial neoplasm of low malignant potential; *LG-UC*, low grade urothelial carcinoma; *HG-UC*, high grade urothelial carcinoma; *Cis*, carcinoma in situ*.*

### Ethics approval and consent to participate

Ethical approval for this project was obtained from the CPP–Est II, the ethics committee of our institution. All procedures performed in this study involving human participants were thus in accordance with the ethical standards of the institutional and/or national research committee and with the 1964 Helsinki declaration and its later amendments or comparable ethical standards. We confirm that informed consent was obtained from all subjects and/or their legal guardian(s) to participation in the study.

### Immunohistochemistry

Tissue samples, obtained by endoscopic resection or cystectomy (partial or total), were fixed in 4% formalin and paraffin-embedded. The tissue blocks were cut serially at 3 μm thickness, deparaffinized with toluene, and rehydrated in graded concentration of ethanol solutions. Antigen retrieval was performed by incubation of sections in 0.5% H_2_O_2_ for 30 min, followed by unmasking in 0.01 mol/L sodium citrate buffer (pH 6.0) for 20 min at high temperature. After non-specific site blocking with 0.5% BSA (bovine serum albumin) for 15 min, the sections were further incubated with primary antibodies (rabbit anti-human E-FABP/FABP5, dilution 1:500, Abcam ab37267; rabbit anti-human A-FABP/FABP4, dilution 1:1500, Abcam ab13979) (Abcam, Paris, France) for one hour at room temperature using the automated IHC/ISH slide staining BenchMark XT instrument (Roche Diagnostics) according to the manufacturer’s instructions. The sections were then washed three times with PBS 1X and incubated for 30 min with the ImmPRESS™ HRP Universal Antibody (anti-mouse IgG/anti-rabbit IgG, peroxidase) (Vector Laboratories, CliniSciences, Nanterre, France). Endogenous peroxidase activity was removed by dipping the sections in 5% H_2_O_2_ for 10 min at room temperature followed by incubation with streptavidin–horseradish peroxidase for 25 min. Finally, peroxidase activity was revealed by DAB (3,3′-diaminobenzidine) staining (0.9 mg/mL) for 7 min. Sections were counterstained with Harris haematoxylin/eosine/safran (HES) with Leica Autostainer XL (Leica Biosystems, Nanterre, France), dehydrated in alcohol, and mounted on cover slips using a standard procedure. Negative controls were obtained by omitting the first antibody. Normal bladder specimens were obtained from patients who had undergone cystoprostatectomy for prostate carcinoma. Control tissues were used for E-FABP staining such as brain as a negative control and psoriatic skin as a positive control. The status of E-FABP and A-FABP was assessed in a coded manner by a pathologist without knowledge of the clinical or pathological features of the patient. For each section, the presence of E-FABP and A-FABP immunostaining in endothelial cells was checked as an internal control. The proportion of stained cells was used as a criterion of evaluation. E-FABP staining was considered high when > 30% of cells were moderately or strongly stained, and low when staining was weak or when ≤ 30% of cells were moderately or strongly stained. A-FABP staining was considered positive when > 10% of cells were moderately or strongly stained, and negative when staining was weak or when ≤ 10% of cells were moderately or strongly stained.

### Statistical analyses

Continuous variables were expressed as mean (standard deviation) values and categorical variables as numbers and percentages. Association between clinical and immunohistochemistry data were analyzed using the χ^2^ test or Fisher’s exact test, if appropriate. Means were compared using the Student *t* test. Data from the immunohistochemical expression of E-FABP and A-FABP on the same tumor were compared with McNemar’s test. Overall survival (OS) was calculated from the date of diagnosis to the death from any cause. Alive patients were censored at the last follow-up. Recurrence-free survival (RFS) was defined as the interval between the date of TUR and the date of the first recurrence. Progression-free survival (PFS) was defined as the interval from the date of diagnosis to the earliest date of disease progression (local and distant). OS and PFS were estimated using Kaplan–Meier method and described using median (or rate at specific time points) with 95% confidence interval (CI). Survival curves were compared using log-rank test. Follow-up was calculated using reverse Kaplan–Meier estimation. The estimation of parameters with OS was first assessed using log-rank test and then included (for those with *p* < 0.05) in a final multivariate Cox regression model with stepwise backward elimination. All statistical tests were two-sided, and *p* < 0.05 was considered statistically significant. Data were analyzed by using PASW Statistics for Windows, Version 18.0. Chicago: SPSS Inc.

## Results

### Patient characteristics

A cohort of 210 patients including 171 men (81.43%) and 39 women (18.57%) with a sex ratio of 4.4 was investigated. The mean age was 70.7 years (median 72, range 25–99) with a standard deviation of 12.9 years. The median follow-up was 172.7 months [162.7;182.7]. The histopathological stage examination showed 80 pTa, 75 pT1 and 55 pT2–T4. The subtype of 191 (91.38%) tumors was the common papillary histological type. The other subtypes were represented by UC with squamous, glandular, or neuroendocrine differentiation, as well as the micropapillary, reversed, and sarcomatoid variants. It should be noted that “histology” information is missing for a patient. Grading according to 2004 WHO/ISUP was the following: 18 PUNLMP (papillary urothelial neoplasm of low malignant potential), 48 LG-UC (low grade UC), 14 HG-UC (high grade UC) for pTa, 2 PUNLMP, 9 LG-UC, 64 HG-UC for pT1 and 3 LG-UC, 52 HG-UC for pT2–T4. The presence of C*is* (carcinoma in situ) was diagnosed in 10 cases (4.78%). It should be noted that the presence or not of a C*is* is missing for a patient.

### Immunohistochemical assessment of E-FABP expression

**Figure 1:**
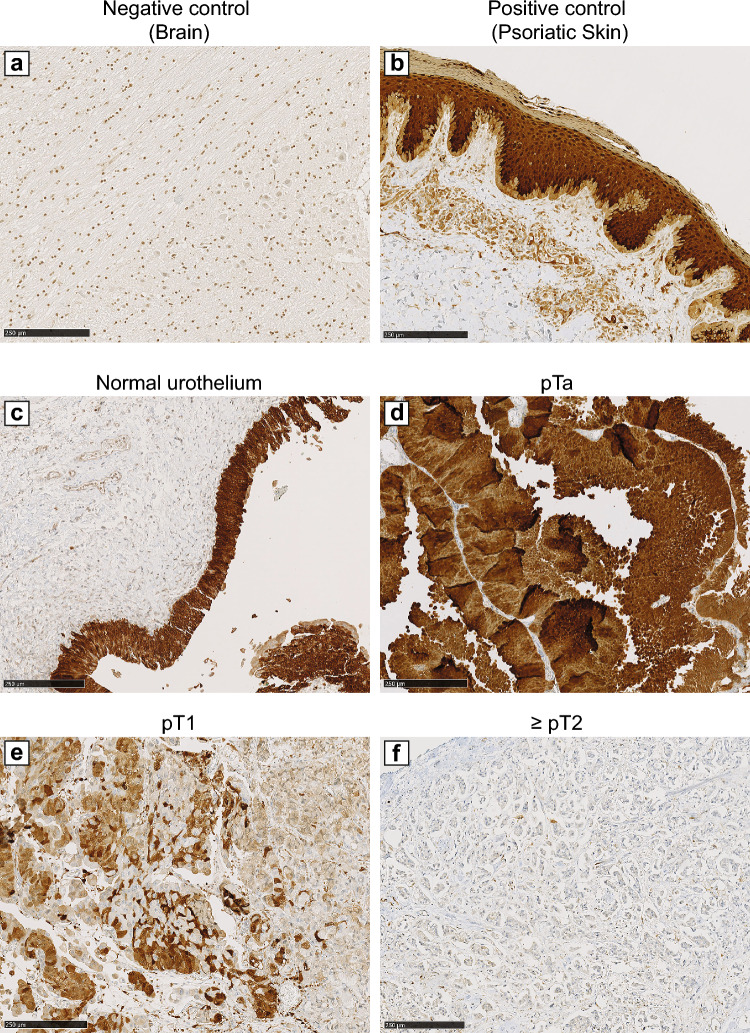
Immunohistochemical analysis of E-FABP expression in brain (negative control) (**a**), psoriatic skin (positive control) (**b**), normal urothelium (**c**), pTa papillary UC (**d**), pT1 (**e**) and pT2 tumors (**f**). Magnification X200.

The expression pattern of E-FABP was examined by immunohistochemistry on 210 tumor sections. The results of E-FABP immunostaining are summarized in Table 1. Of 210 UC samples, 163 (77.62%) presented a high expression of E-FABP and 47 (22.38%) a low expression. The Fig. 1 shows a representative E-FABP immunostaining result of the whole tumors and the control tissues. E-FABP was absent in brain (chosen as a negative control) (Fig. 1a). On the other hand, as expected, it was detected in a psoriatic skin specimen, used as a positive control (Fig. 1b). E-FABP expression was highly expressed in the entire height of the normal urothelium (Fig. 1c). Importantly, a decreased expression of the protein was observed according to the pathologic stage (Fig. 1d,e,f). More specifically, a high E-FABP expression was detected both in normal urothelium and in pTa tumors, it decreased in pT1 tumors and was very low in pT2 tumors.

### Association of E-FABP expression with clinicopathological parameters

Putative association between E-FABP expression and clinical data was analyzed (Table 1). A positive E-FABP staining was detected in 93.75% pTa, 77.33% pT1 and 54.54% pT2-T4. The diminution of 30 to 40% of the E-FABP expression is in line with the previously described immunohistochemical results (Fig. 1). In addition, 95% PUNLMP, 93.33% LG-UC and 67.70% HG-UC were E-FABP positive (high expression). A statistically significant association between the decreased expression of E-FABP and tumor stage (*p* < 0.001) and histological grade (*p* < 0.001) was demonstrated. The presence of metastatic lymph nodes as well as the presence of visceral metastases were also correlated with the diminution of E-FABP expression (*p* ≤ 0.001). On the other hand, there was no association between E-FABP expression and sex, age of the patient, histological type or the presence of associated C*is*. In conclusion, the overall results showed that E-FABP significantly decreased from pT1 tumors and in high grade tumors and a low E-FABP immunostaining was also significantly associated with lymph node status and the presence of metastases.

### Association of clinicopathological characteristics and E-FABP expression with RFS and PFS in pTa and pT1 tumors

**Figure 2:**
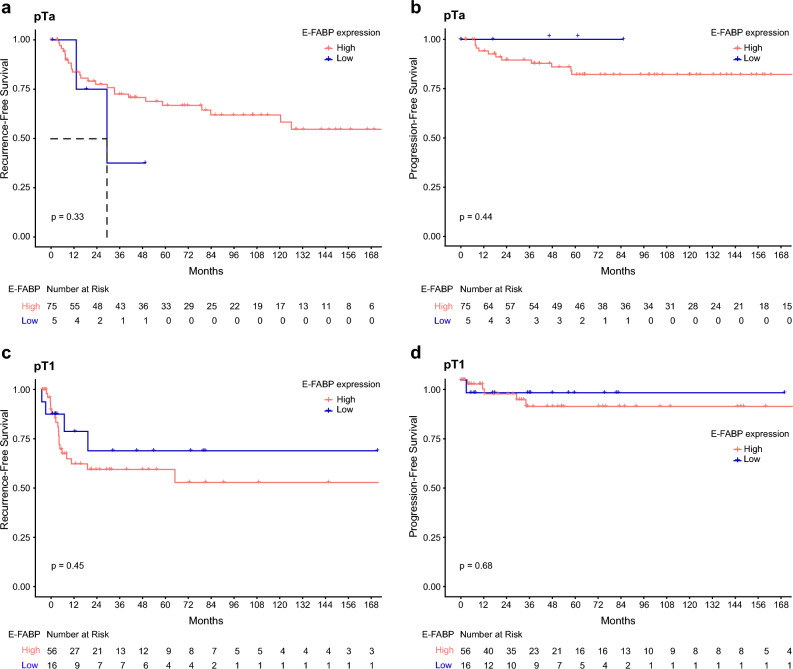
Kaplan–Meier curves for high and low E-FABP expression in relation to RFS and PFS in patients with pTa (**a**, **b**) or pT1 tumor (**c**, **d**).

A univariate survival analysis was performed in pTa and pT1 tumors to study RFS and PFS and their correlation with clinical and histological parameters as well as with the expression of E-FABP. No significant correlation was observed between clinical data, E-FABP expression and RFS as well as PFS (Fig. 2). To conclude, despite the promising results of the immunohistochemical analysis, our results showed that a low expression of E-FABP (≤ 30% stained cells) has no prognostic or predictive value in NMIBC for RFS and PFS.

### Association of clinicopathological parameters and E-FABP expression with OS

**Figure 3:**
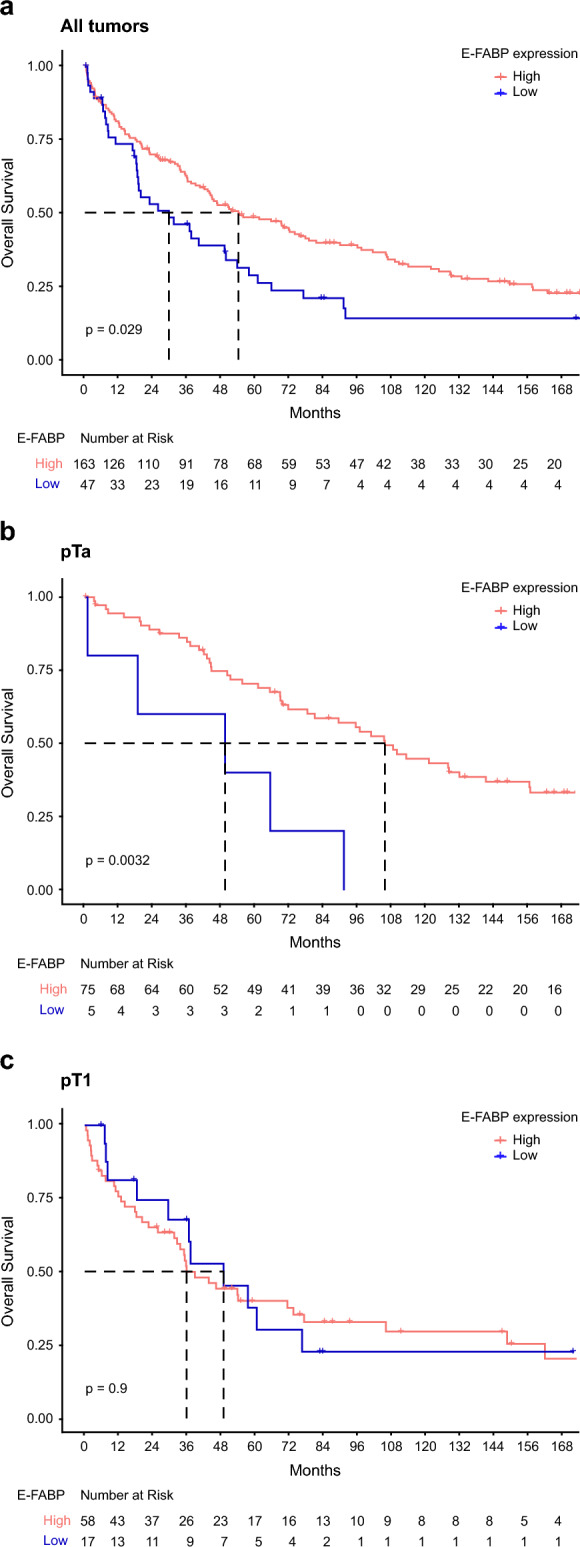
Kaplan–Meier plots comparing overall survival for patients stratified by E-FABP expression level in the whole cohort (**a**), in patients with pTa (**b**) or pT1 (**c**) UC.

As previously, a univariate statistical analysis was carried out in the overall cohort of patients with UC as well as in pTa and pT1 groups to correlate OS with different clinical and histological parameters and with E-FABP expression (Table 2, Fig. 3). According to the results obtained, older patients (> 72 years old) had a shorter median OS (mOS) than younger patients (35.1 months vs. 69.0 months; *p* < 0.001). Patients with a papillary tumor had a longer mOS (51.3 months vs. 17.1 months for patients with an histological variant; *p* = 0.002). A shorter mOS was also associated with high stage pT2-T4 (29.3 months vs. 95.7 months for pT1 and 100.9 months for pTa; *p* < 0.001), the presence of metastatic lymph nodes (13.9 months vs. 55.5 months; *p* < 0.001), the presence of metastases (3.3 months vs. 54 months; *p* < 0.001), and a high grade (29.3 months vs. 95.7 months for LG-UC and 100.9 months for PUNLMP; *p* < 0.001). Interestingly, patients presenting UC with more than 30% positive cells for E-FABP (high E-FABP expression) had a longer mOS (53.8 months vs. 29.3 months; *p* = 0.029). Thus, the expression of E-FABP above a 30% threshold in UC could be an indicator of a good prognosis for the clinical outcome of patients with NMIBC who will progress or not. A multivariate analysis was carried out including the significant parameters of the univariate analysis, i.e. age, histology, stage, lymph node status, metastatic status, grade as well as E-FABP expression. The multivariate Cox model showed that median age (> 72 years), stage and metastatic status were independent prognostic factors (*p* < 0.001 for the three parameters). But, E-FABP expression was not an independent significant prognostic marker in the overall cohort and in the pT1 group. In contrast, it was an independent prognostic marker in the pTa group (*p* = 0.02).

**Table 2:** Univariate and multivariate analyses of clinicopathological data and E-, A-FABP expression in relation to OS of patients.

All tumors									pTa								pT1			
Parameters	Univariate analysis				Multivariate analysis				Univariate analysis				Multivariate analysis				Univariate analysis			
	mOS	95% CI		*p* value	HR	95% CI		*p* value	mOS	95% CI		*p* value	HR	95% CI		*p* value	mOS	95% CI		*p* value
		** − **	** + **			** − **	** + **			** − **	** + **			** − **	** + **			** − **	** + **	
Sex																				
Male	54	38.2	69.9	0.185					157.5	74.8	240.2	0.26					53.8	33.5	77.4	0.16
Female	35.6	16.6	54.6						95.7	59.2	162.2						35.6	12.6	NC	
Median Age																				
≤ 72 years	69.0	26.5	111.5	** < 0.001**	0.5	0.3	0.6	** < 0.001**	121.6	56.9	186.2	** < 0.01**	0.5	0.2	0.7	**0.01**	149.3	34.9	NC	** < 0.01**
> 72 years	35.1	24.6	45.6		Ref				81.2	56.5	105.8		Ref				35.6	2.9	54	
Histology																				
Papillary	51.3	7.1	27.1	**0.002**					101.1	72.8	129.4	0.81					–			
Variants	17.1	40.0	62.6						91.5	NC	NC						–			
pT																				
pTa	100.9	40.4	161.4	** < 0.001**	0.4	0.2	0.6	** < 0.001**	–								–			
pT1	95.7	64.7	126.7		0.3	0.4	0.9	**0.02**	–								–			
pT2-4	29.3	19.4	39.2		Ref				–								–			
pN																				
0	55.5	41.1	69.9	** < 0.001**					–								–			
1–2	13.9	1.7	26.1						–								–			
pM																				
0	54.0	38.0	69.9	** < 0.001**	0.3	0.1	0.5	** < 0.001**	–								–			
1	3.3	0.0	10.2		Ref				–								–			
Grade																				
PUNLMP	100.9	40.4	161.4	** < 0.001**					89.6	5.1	174.1	0.83					NR			0.53
LG-UC	95.7	64.7	126.7						105.7	89.7	121.7						77.4	2	NC	
HG-UC	29.3	19.4	39.2						51.3	0.1	102.4						37.2	31.4	60.6	
A-FABP expression																				
Negative	31.4	19.5	43.3	** < 0.001**					95.7	76.8	111.6	0.74					32.4	15	49.8	0.84
Positive	81.2	45.3	117.1						108.9	51.5	166.3						71.5	36.4	106.6	
E-FABP expression																				
Low	29.3	7.5	51.1	**0.029**					49.4	0	115.7	** < 0.01**	Ref				48.9	24.8	73	0.90
High	53.8	34.7	72.9						106	86.3	125.7		0.3	0.1	0.8	**0.02**	35.8	22.8	48.8	
A/E-FABP expression																				
Negative/Low	19.4	9.5	29.3	** < 0.01**					49.4	0	115.7	**0.01**					36.6	19.5	53.7	0.39
Positive/High	105.7	51.9	159.5						NR								71.5	6.2	136.8	
Negative/High	37.1	21.7	52.5						108.9	51.5	166.3						31.4	8	54.8	
Positive/Low	57.5	19.0	96.0						101.1	83.6	128.4						57.5	24.1	90.7	

*OS*, overall survival; *PUNLMP*, papillary urothelial neoplasm of low malignant potential; *LG-UC*, low grade urothelial carcinoma; *HG-UC*, high grade urothelial carcinoma; *Cis*: carcinoma in situ; *HR*, hazard ratio; *NR*, not reached; *NC*, not calculable.

Significant values are in bold.

### Status of E-FABP expression in A-FABP-negative tumors

In a previous work, we showed a loss of A-FABP expression in high stage/grade UC^10^. Furthermore, a study, carried out in adipose tissue of A-FABP null mice, revealed an increase of E-FABP expression to compensate the absence of A-FABP^13^. In this context, since only E- and A-FABP are expressed in bladder cancers, we investigated whether there was a gain of E-FABP expression in UC for which A-FABP was lost. Thus, the expression of E-FABP was investigated within A-FABP negative patient population (Table 3). The analysis of A-FABP expression in our cohort of 210 patients showed that 42.50% pTa were negative, as well as 57.33% pT1 and 92.72% pT2-T4 (data shown in Tables 1 and 3). In the A-FABP-negative pTa cases, 85.30% presented a high E-FABP expression. In the pT1 group, 74.42% cases had a high E-FABP level and in the pT2-T4 group, 54.90% cases still had a high expression of E-FABP although E-FABP level decreased depending on the stage (Table 3). Maintaining a high expression of E-FABP in MIBC may counterbalance the loss of A-FABP expression in this group of tumors.

**Table 3:** E-FABP expression analysis in pTa, pT1, pT2-T4 groups according to the A-FABP status (negative or positive immunodetection).

**pTa** (80)		**A-FABP expression, n (%)**		***p*** **value**
		**Negative (≤ 10%)**	**Positive (> 10%)**	
		34 (42.50)	46 (57.50)	
**E-FABP expression, n (%)**	Low	5 (14.70)	0 (0.00)	0.03
	High	29 (85.30)	46 (100.00)	
**pT1** (75)		**A-FABP expression, n (%)**		
		**Negative (≤ 10%)**	**Positive (> 10%)**	
		43 (57.33)	32 (42.66)	
**E-FABP expression, n (%)**	Low	11 (25.60)	6 (18.75)	0.07
	High	32 (74.42)	26 (81.25)	
**pT2-pT4** (55)		**A-FABP expression, n (%)**		
		**Negative (≤ 10%)**	**Positive (> 10%)**	
		51 (92.72)	4 (7.27)	
**E-FABP expression, n (%)**	Low	23 (45.10)	2 (50.00)	1
	High	28 (54.90)	2 (50.00)	

### Status of E-FABP expression in A-FABP-positive tumors

As presented in Table 3, it worth be noting that in the pTa group presenting a A-FABP positive expression, all cases had a high E-FABP expression. In the A-FABP positive pT1 group, for which A-FABP decreased compared to pTa tumors, 81.25% cases had a high E-FABP expression. In pTa-pT1 tumors, we notice that E-FABP was highly expressed. In pT2-T4 group, very few cases were A-FABP positive (4/55 cases) which was consistent with our previously published results^10^.

### Overall survival analysis according to E- and A-FABP status

**Figure 4:**
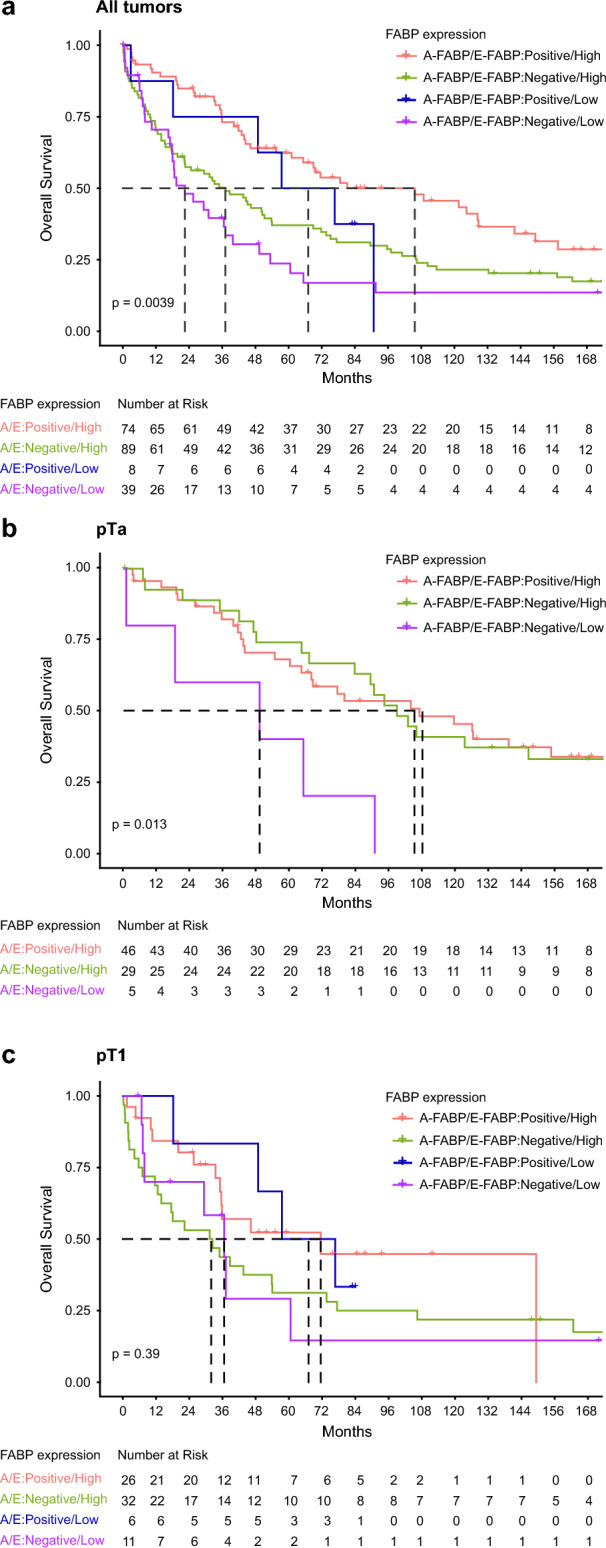
Kaplan–Meier curves comparing overall survival for patients stratified according to both E- and A-FABP expression status in the whole cohort (**a**), in patients with pTa (**b**) or pT1 (**c**) UC.

Kaplan–Meier curves, plotted according to the status of E- and A-FABP in the overall cohort and in pTa or pT1 group, are presented in the Fig. 4. All patients with UC or patients with a pTa tumor presenting a low expression of E- and A-FABP, exhibited a poorer survival than those with a high expression of both markers (log-rank test *p* = 0.0039 and *p* = 0.013 respectively). For patients with a pT1 tumor, the association of E- and A-FABP status was not significantly associated with OS. To conclude, there was a significant association between E- and A-FABP in the cohort of UC and pTa population. The patients who maintained the expression of both markers had a better survival, those with the negative expression of a single marker had an intermediate survival value. Nevertheless, it seemed better to still express A-FABP in terms of survival.

## Discussion

NMIBC represent the majority of new diagnosed UC. According to the guidelines of the European Association of Urology (EAU), they are classified as follows: low, intermediate, and high risk UC. To date, urologists cannot predict the clinical course of patients with a high risk NMIBC after resection. To better select these patients, tissue biomarkers are needed because UC lack a predictive molecular marker of progression used in clinical practice^15^. Currently, the presence of C*is*, the high grade pT1 tumors, the multifocality of carcinomas and the lymph node status remain the significant predictive factors of progression collectively accepted by the clinicians^16,17^. Analyses performed by many researchers allowed to identify some potential prognostic factors from tissue or liquid biopsies (urine, blood) in order to assess risks of tumor progression. Circulating biomarkers such as ctDNA (circulating tumor DNA), CTC (circulating tumor cell) and exosomes seem promising for cancer in general because they are non-invasive and reflect genetic and epigenetic heterogeneity between patients. Nevertheless, they show a low sensitivity for NMIBC leading to their limitation for their use in clinical routine^18^. Cytokines (IL-2), various immunological (CD4 + and GATA3 + T cells, tumor associated macrophages) and genetic biomarkers have been examined and could be used as a means to predict NMIBC clinical course^19^.

In a previous work, we found that loss of A-FABP protein can influence tumor progression of NMIBC^10^. In the present study, we focused on another member of the FABP family, the E-FABP because it is the only FABP, with A-FABP, expressed in bladder cancers. Following an immunohistochemical analysis, we confirmed the high expression of E-FABP in healthy urothelium as previously described^20^. We demonstrated that loss of this protein was correlated with high tumor stage and histologic grade as well as the presence of metastatic lymph nodes and visceral metastases. Unfortunately, when conducting a long-term study on patient outcomes, the decreased expression of E-FABP in the resected primary tumor was not a risk factor of recurrence or progression. However, the univariate survival analysis showed that the high expression of E-FABP (> 30%), was associated with a better prognosis for OS in patients of the overall cohort and in patients with pTa tumor. Thus, the presence of E-FABP was predictive to the absence of death. But we failed to identify E-FABP as an independent factor in the multivariate analysis.

Few works have investigated the expression of E-FABP in bladder cancer. A study reported that the expression of the *FABP5* gene was significantly higher in NMIBC specimens than in normal bladder samples^21^. Furthermore, less-differentiated bladder tumors were characterized by a decreased expression of E-FABP protein^12^ and low-grade tumors expressed E-FABP to a higher extent than their high-grade counterparts^20^. In the same way, Celis and his colleagues investigated normal urothelial tissue and UC (grade III, T2-4) samples and found that expression of E-FABP was downregulated in invasive UC^22,23^. A protein analysis by two-dimensional gel electrophoresis and mass spectrometry revealed that the down-regulated spots (spots 1–7) in grade III NMIBC were identified as FABP5^24^.

E-FABP is expressed in a large number of human tissues and consequently it is deregulated in cancers from different origin. Many studies reported contrary results with an up-regulation or a down-regulation of E-FABP in different cancer models.

However, an increase of E-FABP is much more described. Thus, several studies have reported that FABP5 was upregulated in cholangiocarcinoma and hepatocellular carcinoma, prostatic carcinoma, glioma, oral squamous cell carcinoma, cervical cancer, colorectal cancer, pancreatic cancer, non-small cell lung cancer, breast cancer^25^. Elevated expression of E-FABP has been correlated with poor prognosis in vulvar carcinoma^26^, cervical cancer^27^, hepatocellular carcinoma^28^, clear renal cell carcinoma^29^ and uveal melanoma^30^. But the molecular mechanisms connecting E-FABP and cancer biology remain undefined. However, FABP5 binds and transports long-chain fatty acids, and some of them could be implied as signaling molecules in carcinogenesis process^31^. As an important carrier of fatty acids, elevated expression of FABP5 could increase their signaling activity. Most in vitro studies associated the upregulation of E-FABP with an increased cell proliferation, cell migration and invasiveness in several cancers, such as oesophageal cancer or breast cancer^32,33^. E-FABP could act on tumor microenvironment through the upregulation of VEGF^34^, the increase of MMP9 to provide an invasive phenotype^35^ and the stimulation of cancer progression and metastasis through the epithelial-mesenchymal transition process^36^.

Experimental assays performed to decrease E-FABP expression validate the contribution of this FABP to cell proliferation, cell migration and metastatic process. Thus, the highly invasive PC-3 M prostate cancer cell line transfected by a vector expressing an antisense E-FABP transcript exhibited less invasive capacities in vitro in comparison with the mock transfected cells. The injection of these E-FABP-deficient PC-3 M cells into a nude mouse model produced tumors with a reduced size compared to those produced by mock transfected cells and this was associated with a decrease of VEGF expression and microvessel density^37^. Furthermore, the downregulation of FABP5 has the potential to inhibit cervical cancer cell proliferation, cell migration, colony formation and invasion capacity in vitro and to decrease tumor growth, to suppress metastasis activity in mouse. This was associated with the reduction of MMP2 and MMP9 expression^27^. In addition, *FABP5* gene silencing by RNA interference technology inhibited the proliferation and invasive properties of human SGC‑7901 gastric cancer cells in vitro and triggered a G0/G1 cell cycle arrest leading to apoptosis^38^.

By contrast, few publications reported a decreased expression of E-FABP in different models. Thus, E-FABP expression was down-regulated in breast cancer cell lines in comparison with breast normal cell lines^39^. It was identified as a cDNA down-regulated in nodal metastasis relative to the primary breast carcinoma^40^. The urinary test for E-FABP in patients with cutaneous melanoma revealed that the protein was absent in patients with distant metastasis (stage IV) indicating an inverse relationship between E-FABP release and the dissemination of melanoma^41^. A northern blotting analysis disclosed that E-FABP expression was higher expressed in the primary squamous cell carcinoma of the oral tongue (67%) than in the corresponding metastatic lymph nodes^42^. To our knowledge, apart from the present work, only one study, carried out by a two-dimensional PAGE protein analysis, reported that less-differentiated bladder squamous cell carcinomas showed a reduced expression of E-FABP in comparison with their more differentiated counterparts^12^.

To date, the molecular mechanisms involved in the decrease of E-FABP expression in bladder cancer have not to be elucidated. Several hypotheses can be put forwards such as a polymorphism or epigenetic modifications. But to our knowledge, no data from the scientific literature were available on these hypothetic mechanisms.

In a previous study, we showed that loss of A-FABP/FABP4 was an indicator of progression for pTa/pT1 UC^10^. In the present work, we reported through a univariate analysis that the high expression of E-FABP was associated with a better prognosis for OS across the overall UC cohort and the pTa group and thus was predictive to the absence of death. Thus, as a result of our studies, we showed, from the same paraffin-embedded sections, that E-FABP and A-FABP were deregulated in UC. Simultaneous analysis of both markers showed in NMIBC with negative A-FABP that E-FABP expression remained at high levels. Thus, maintaining high E-FABP expression appears to counteract A-FABP decrease in these tumors. On the other hand, in MIBC with negative A-FABP (92.72% cases), it still remained 54.90% cases with a high E-FABP expression. Nevertheless, E-FABP expression was lower in MIBC than in NMIBC. These results suggested that E-FABP appears to compensate for the lack of A-FABP except in high stage UC. Furthermore, the combined analysis of both proteins correlated with more accuracy with grade and/or stage of the disease and predicted a worse prognosis for patients. Indeed, the patient group with negative A-FABP/low E-FABP had a shorter OS than those with positive A-FABP/high E-FABP. However, it should be noted that for the pTa tumor group, there are only 5 patients with negative A-FABP and low E-FABP. This is significant, but there are not enough cases to conclude. For the other pTa, A-FABP expression is positive and E-FABP is highly expressed. However, for the pT1 tumor group, when A-FABP is negative, E-FABP remains present, with many tumors still expressing E-FABP. Indeed, E-FABP tries to compensate for the loss of A-FABP. It is therefore important to carry out simultaneous immunohistochemical analysis of both proteins, rather than E-FABP alone. It is assumed that a single marker will not have the sensitivity and specificity required on its own to diagnose UC and prognosticate their clinical evolution.

For future perspectives, we could hypothesize that the overexpression of both E- and A-FABP could block tumor progression and consequently improve patient survival. Interestingly, these proteins are direct target of the nuclear receptor PPARβ. More precisely, transcriptional activation of PPARβ increases E-FABP in prostate cancer cells^43^ and we show in a previous work that it raises A-FABP in T24 bladder cancer cells^44^. Therefore, intravesical instillations of PPARβ agonists could be an attractive therapeutic strategy acting simultaneously on both markers for a better management of patients.

## Conclusion

For the first time, we show that the concomitant immunostaining evaluation of A- and E-FABP allowed us to better stratify the patients with UC for optimizing treatment and follow-up.

## Data Availability

All data generated or analyzed during this study are included in this published article.
